# Observation Necessary in a Physician*From *Mat. Med. Pura*, vol. iv, 2d edit., 1825.

**Published:** 1883-10

**Authors:** Samuel Hahnemann


### OBSERVATION NECESSARY IN A PHYSICIAN.*

Samuel Hahnemann.

In order to be able to observe well, the medical practitioner requires to possess, what is not to be met with among ordinary physicians even in a moderate degree, the capacity and habit of noticing carefully and correctly the phenomena that takes place in natural diseases, as well as those that occur in the morbid states artificially excited by medicines when they are tested upon the healthy body, and the ability to describe them in the most appropriate and natural expressions.

In order accurately to perceive what is to be observed in patients, we should direct all our thoughts upon the matter we have on hand, come out of ourselves, as it were, and fasten ourselves, so to speak, with all our powers of concentration upon it, in order that nothing that is actually present, that has to do with the subject, and that can be ascertained by all the senses, may escape us.

Poetic fancy, fantastic wit and speculation, must for the time be suspended, and all overstrained reasoning, forced interpretation, and tendency to explain away things must be suppressed. The duty of the observer is only to take notice of the phenomena and their course; his attention should be on the watch, not only that nothing actually present escape his observation, but that also what he observes is understood exactly as it is.

This capability of observing accurately is never quite an innate faculty; it must be chiefly acquired by practice, by refining and regulating the perceptions of the senses, that is to say, by exercising a severe criticism in regard to the rapid impressions we obtain of external objects, and at the same time the necessary coolness, calmness, and firmness of judgment must be preserved, together with a constant distrust of our own powers of apprehension.

---

* From *Mat. Med. Pura*, vol. iv, 2d edit., 1825.

The vast importance of our subject should make us bestow the energies of our body and mind upon the observation; and great patience, supported by the power of the will, must sustain us in this direction until the completion of the observation.

To educate us for the acquirement of this faculty, an acquaintance with the best writings of the Greeks and Romans is useful, in order to enable us to attain directness in thinking and in feeling, as also appropriateness and simplicity in expressing our sensations; the art of drawing from nature is also useful, as it sharpens and practices our eye, and thereby also our other senses, teaching us to form a true conception of objects, and to represent what we observe, truly and purely, without any addition from the fancy. A knowledge of mathematics also gives us the requisite severity in forming a judgment.

Thus equipped, the medical observer cannot fail to accomplish his object, especially if he has at the same time constantly before his eyes the exalted dignity of his calling—as the representatives of the all-bountiful Father and Preserver, to minister to His beloved human creatures by renovating their systems when ravaged by disease. He knows that observations of medical subjects must be made in a sincere and holy spirit, as if under the eye of the all-seeing God, the Judge of our secret thoughts, and must be recorded so as to satisfy an upright conscience, in order that they may be communicated to the world, in the consciousness that no earthly good is more worthy of our zealous exertions than the preservation of the life and health of our fellow-creatures.

The best opportunity for exercising and perfecting our observing faculty is afforded by instituting experiments with medicines upon ourselves. Whilst avoiding all foreign medicinal influences and disturbing mental impressions in this important operation, the experimenter, after he has taken the medicine, has all his attention strained toward all the alterations of health that take place on and within him, in order to observe and correctly to record them, with ever-wakeful feelings, and his senses ever on the watch.

By persevering in this careful investigation of all the changes that occur within and upon himself, the experimenter attains the capability of observing all the sensations, be they ever so complex, that he experiences from the medicine he is testing, and all, even the finest shades of alteration of his health, and of recording in suitable and adequate expressions his distinct conception of them.

Thus only is it possible for the beginner to make pure, correct, and undisturbed observations, for he knows that he will not deceive himself, there is no one to tell him aught that is untrue, and he himself feels, sees, and notices what takes place in and upon him. He will thus acquire practice to enable him to make equally accurate observations on others also.

By means of these pure and accurate investigations we shall be made aware that all the symptomatology hitherto existing in the ordinary system of medicine was only a very superficial affair, and that nature is wont to disorder man in his health and in all his sensations and functions by disease or medicine in such infinitely various and dissimilar manners, that a single word or a general expression is totally inadequate to describe the morbid sensations and symptoms, which are often of such a complex character, if we wish to portray really, truly, and perfectly the alterations in the health we meet with.

No portrait painter was ever so careless as to pay no attention to the marked peculiarites in the features of the person he wished to make a likeness of, or to consider it sufficient to make any sort of a pair of round holes below the forehead by way of eyes, between them to draw a long-shaped thing directed downward, always of the same shape, by way of a nose, and beneath this to put a slit going across the face, that should stand for the mouth of this or of any other person; no painter, I say, ever went about delineating human faces in such a rude and slovenly manner; no naturalist ever went to work in this fashion in describing any natural production; such was never the way in which any zoologist, botanist, or mineralogist acted.

It was only the semeiology of ordinary medicine that went to work in such a manner, when describing morbid phenomena. The sensations that differ so vastly among each other, and the innumerable varieties of the sufferings of the many different kinds of patients, were so far from being described by word or writing according to their divergences and varieties, according to their peculiarities; the complexity of the pains composed of various kinds of sensations, their degrees and shades, was so far from being accurately or completely described, that we find all these infinite varieties of sufferings huddled together under a few bare, unmeaning, general terms, such as *perspiration*, *heat*, *fever*, *headache*, *sore throat*, *croup*, *asthma*, *cough*, *chest complaints*, *stitch in the side*, *belly-*

*ache, want of appetite, indigestion, dyspepsia, backache, coxalgia, hæmorrhoidal sufferings, urinary disorders, pains in the limbs* (called according to fancy *gouty* or *rheumatic*), *skin diseases, spasms, convulsions,* etc. With such superficial expressions, the innumerable varieties of sufferings of patients were disposed of in the so-called observations, so that—with the exception of some one or other severe striking symptom in this or that case of disease—almost every disease pretended to be described is as like another as the spots on a die, or as the various pictures of the dauber resemble one another in flatness and want of character.

The most important of all human vocations, I mean *the observation of the sick, and of the infinite varieties of their disordered state of health,* can only be pursued in such a superficial and careless manner *by those who despise mankind,* for in this way there is no question either of distinguishing the peculiarities of the morbid states or of selecting the only appropriate remedy for the special circumstances of the case.

The conscientious physician who earnestly endeavors to apprehend in its peculiarity the disease to be cured, in order to be able to oppose to it the appropriate remedy, will go much more carefully to work in his endeavor to distinguish what there is to be observed; language will scarcely suffice to enable him to express by appropriate words the innumerable varieties of the symptoms in the morbid state; no sensation, be it ever so peculiar, will escape him, which was occasioned in his feelings by the medicine he tested on himself; he will endeavor to convey an idea of it in language by the most appropriate expression, in order to be able in his practice to match the accurate delineation of the morbid picture with the similarly acting medicine, whereby alone, as he knows, can a cure be effected.

So true it is that the careful observer alone can become a true healer of diseases.

